# Forty years after the first totally implantable venous access device (TIVAD) implant: the pure surgical cut-down technique only avoids immediate complications that can be fatal

**DOI:** 10.1007/s00423-021-02225-6

**Published:** 2021-06-09

**Authors:** Adriana Toro, Elena Schembari, Emanuele Gaspare Fontana, Salomone Di Saverio, Isidoro Di Carlo

**Affiliations:** 1 General Surgery, Augusta Hospital, Syracuse, Italy; 2 Department of General Surgery, Whipps Cross University Hospital-Barts Health NHS Trust, Whipps Cross Rd, Leytonstone, , London, E11 1NR UK; 3 Department of Surgical Sciences and Advanced Technologies “G.F. Ingrassia,” Cannizzaro Hospital, General Surgery, University of Catania, Via Messina 829, 95126 Catania, Italy; 4 Department of General Surgery, University Hospital of Varese, University of Insubria, Varese, Italy; 5 Cambridge Colorectal Unit, Addenbrooke’s University Hospital NHS Foundation Trust, Cambridge, UK

**Keywords:** Totally implantable venous access device (TIVAD), Surgical cut-down, Percutaneous approach, Pneumothorax, Port-a-cath

## Abstract

**Aim:**

Even though TIVADs have been implanted for a long time, immediate complications are still occurring. The aim of this work was to review different techniques of placing TIVAD implants to evaluate the aetiology of immediate complications.

**Methods:**

A systematic literature review was performed using the PubMed, Cochrane and Google Scholar databases in accordance with the PRISMA guidelines. The patient numbers, number of implanted devices, specialists involved, implant techniques, implant sites and immediate complication onsets were studied.

**Results:**

Of the 1256 manuscripts reviewed, 36 were eligible for inclusion in the study, for a total of 17,388 patients with equivalent TIVAD implantation. A total of 2745 patients (15.8%) were treated with a surgical technique and 14,643 patients (84.2%) were treated with a percutaneous technique. Of the 2745 devices (15.8%) implanted by a surgical technique, 1721 devices (62.7%) were placed in the cephalic vein (CFV). Of the 14,643 implants (84.2%) placed with a percutaneous technique, 5784 devices (39.5%) were placed in the internal jugular vein (IJV), and 5321 devices (36.3%) were placed in the subclavian vein (SCV). The number of immediate complications in patients undergoing surgical techniques was 32 (1.2%) HMMs. In patients treated with a percutaneous technique, the number of total complications were 333 (2.8%): 71 PNX (0.5%), 2 HMT (0.01%), 175 accidental artery punctures AAP (1.2%) and 85 HMM (0.6%). No mortality was reported with either technique.

**Conclusion:**

The percutaneous approach is currently the most commonly used technique to implant a TIVAD, but despite specialist’s best efforts, immediate complications are still occurring. Surgical cut-down, 40 years after the first implant, is still the only technique that can avoid all of the immediate complications that can be fatal.

## Introduction

In 1980, Dr. John Niederhuber realized an idea when faced with a family situation. His wife, who was affected by cancer, needed multiple venous infusions that progressively damaged the status of her veins. This inspired the invention of the totally implantable venous access device that was first manufactured by Pharmacia® [1]. Inspired by an act of love, this invention still represents a milestone for oncological patients. It mitigates the local toxicity of antineoplastic drugs, shortens the length of the infusions and undoubtedly improves the quality of life of the patients. In addition to patients with cancer, many categories of patients needing continuous venous catheterizations use these devices. Since the first implant, the Seldinger technique has been used for 39 years [2], but Dr. Niederhuber chose a surgical technique using a small peripheral vein to place his catheter in the vena cava [3]. No percutaneous technique was utilized for the first 10 years, and no immediate lethal complications have been reported.

In the last 30 years, the percutaneous technique has spread worldwide to satisfy the increasingly numerous requests for TIVAD implants. In this way, many physicians, not surgeons, such as anaesthesiologists and radiologists, have applied the easiest and most available Seldinger technique to perform TIVAD implantation. This technique, which usually does not need an operating room, has the disadvantage of immediate complications, such as pneumothorax, haemopneumothorax, arterial puncture and haematoma. Despite these risks, the incessant requests over the years have made the percutaneous technique the technique of choice rather than surgical cut-down. In association with the massive diffusion of percutaneous techniques worldwide, its related complications have also become well-known [4]. Some of these complications are particularly dangerous because they could cause patient death [4].

To reduce the complications of the percutaneous approach during recent decades, many attempts have been made, and the exact identification of the location of the vein before puncture using ultrasound (US) landmark blinding is the most commonly used method [5]. In recent years, improvement of this technique has resulted in real-time ultrasound guidance [6]. However, despite the technological assistance, immediate complications continue to be reported in the literature and reduce the quality of life of the patients, who are usually already sick and fragile [7].

The aim of this work was to explore the progress in relation to all immediate complications following different techniques adopted for placing TIVAD implants in the last 10 years. We limited this study to the last 10 years because in our previous study [8], we evaluated the immediate complications associated with the surgical cut-down and percutaneous approach technique between the first implant and 2010.

## Methods

A systematic literature review was performed using the PubMed, Cochrane and Google Scholar databases, in accordance with the PRISMA guidelines [9] to identify published studies from January 2010 through April 2021.

The keywords used for the search were “totally implantable venous access device”, “totally implantable venous access port”, “port-a-cath”, “percutaneous technique”, “cutdown technique” and “immediate complications”. These keywords were used individually or with the help of the Boolean operator “AND”.

All abstracts were read. Case reports, letters, comments, articles not written in the English language and articles on animals were excluded. Articles analysing adult patients with a TIVAD and immediate complications were considered for full-text review.

We collected articles showing the patient number, number of implanted devices, specialists involved (surgeons (general surgeons, vascular surgeons, thoracic surgeons and cardiovascular surgeons) and other specialists (interventional radiologists, anaesthetists, oncologists, etc.)), the implant technique (surgical or percutaneous technique) and the site of the implant, and the onset of immediate complications such as pneumothorax (PNX), haemothorax (HTM), accidental arterial puncture (AAP), and haematoma (HMM). Finally, we searched for ultrasound (US) use during implantation and the related complications recorded with this approach. Sex, age, type of anaesthesia and device characteristics were not considered for the present study because they did not influence the research and consequently did not influence the results. Documents that clearly did not meet the inclusion criteria were excluded at this stage.

Complications are defined as “immediate” if they occur during the first 24 h after implant placement. Related mortality was investigated.

## Results

**Fig. 1:**
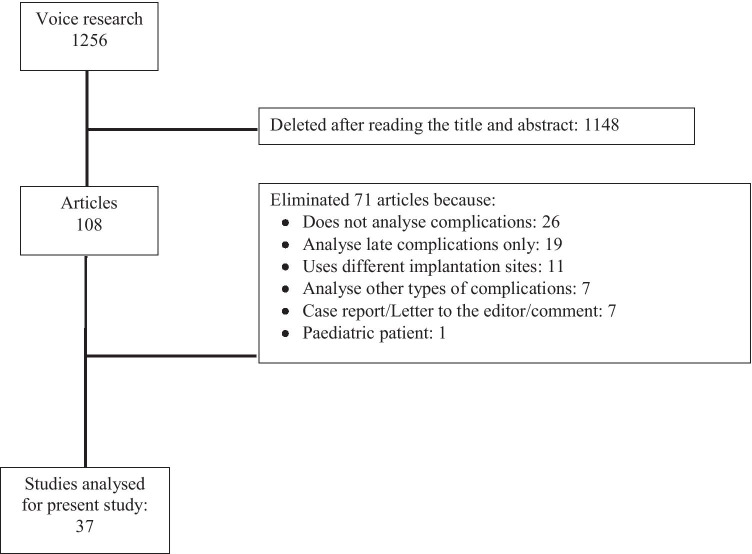
Algorithm used to screen the literature

A total of 1256 manuscripts were examined. After reviewing all of the abstracts, 1148 articles were excluded for not meeting the inclusion criteria. Among the remaining 108 manuscripts, the following were excluded after full-text review: 26 studies because they did not analyse complications; 19 analysed only late complications; 11 used implant sites not compatible with the study; 7 analysed only one type of late complication; 7 were case reports, letters to the editor or a comment; and 1 only analysed paediatric patients (Fig. 1).

Finally, only 37 articles met all of the inclusion criteria and were used for the present study. These manuscripts were published between January 2010 and February 2021.

A total of 17,496 patients received the equivalent number of TIVAD implants, and they were analysed. Of these, 2853 patients (16.3%) were treated with a surgical technique, and 14,643 patients (83.7%) were treated with a percutaneous technique (Tables 1, 2 and 3) [10–46].

**Table 1:** All manuscripts analysed in the present manuscript. Some of these studies [3, 9, 10, 13, 21, 22, 28] reported that surgical cut-down was a percutaneous approach. For this reason, a manuscript with related techniques can be listed in more than one table

Year	Author
2010	Silas [10]
2010	Koketsu [11]
2011	Knebel [12]
2011	Lin [13]
2011	Barbetakis [14]
2011	Di Carlo [15]
2011	Narducci [16]
2012	Kim [17]
2013	Osawa [18]
2015	Lin [19]
2014	Cavallaro [20]
2014	Cheng [21]
2014	Zhou [22]
2014	Granziera [23]
2014	Cavallaro [24]
2014	Seo [25]
2015	Tagliari [26]
2015	Gurkan [27]
2015	An [28]
2015	Wu [29]
2015	Cavallaro [30]
2016	Otsubo [31]
2016	Zerati [32]
2016	Ma [33]
2017	Feo [34]
2017	Seo [35]
2017	Kagawa [36]
2018	Hong [37]
2019	Hashimoto [38]
2019	Sun [39]
2019	Xu [40]
2019	Sun [41]
2019	Sun [42]
2019	Velioglu [43]
2020	Souadka [44]
2020	Mehta [45]
2021	Sun [46]

**Table 2:** Manuscripts with surgical procedures

Year	Author	Number implanted	Surgical technique
2010	Koketsu [11]	74	74
2011	Knebel [12]	53	53
2011	Di Carlo [15]	108	108
2011	Narducci [16]	771	771
2015	Lin [19]	27	27
2014	Cavallaro [20]	155	155
2014	Granziera [23]	102	102
2014	Cavallaro [24]	753	753
2015	Cavallaro [30]	83	83
2016	Otsubo [31]	149	149
2016	Zerati [32]	21	21
2017	Kagawa [36]	254	254
2019	Hashimoto [38]	203	203
2020	Mehta [45]	100	100
		2853	2853

**Table 3:** Manuscripts reporting a percutaneous technique

Year	Author	Number implanted	Percutaneous technique
2010	Silas [10]	536	536
2011	Knebel [12]	49	49
2011	Lin [13]	113	113
2011	Barbetakis [14]	700	700
2012	Kim [17]	441	441
2013	Osawa [18]	207	207
2015	Lin [19]	29	29
2014	Cavallaro [20]	143	143
2014	Cheng [21]	214	214
2014	Zhou [22]	492	492
2014	Granziera [23]	690	690
2014	Seo [25]	216	216
2015	Tagliari [26]	110	110
2015	Gurkan [27]	324	324
2015	An [28]	397	397
2015	Wu [29]	668	668
2016	Otsubo [31]	122	122
2016	Zerati [32]	1202	1202
2016	Ma [33]	2996	2996
2017	Feo [34]	527	527
2017	Seo [35]	932	932
2018	Hong [37]	176	176
2019	Hashimoto [38]	9	9
2019	Sun [39]	55	55
2019	Xu [40]	67	67
2019	Sun [41]	283	283
2019	Sun [42]	619	619
2019	Velioglu [43]	2084	2084
2020	Souadka [44]	135	135
2021	Sun [46]	107	107
		14,643	14,643

Of the 37 manuscripts examined, 15 articles reported the TIVADs were implanted by surgeons, and in 4 of these manuscripts, residents were also involved in the procedures. In 10 manuscripts, the TIVADs were implanted by other physicians (not surgeons), and in 3 of these 10 articles, residents were involved in the procedure. In 11 manuscripts, the physicians that performed the procedure were not specified. Finally, 1 article reported that both surgeons and radiologists performed the procedures. Considering the number of techniques performed in relation to the manuscripts, in the first 14 articles, 8210 procedures were reported, of which 2243 (27.3%) were performed using the cut-down technique and 5967 (72.7%) with percutaneous techniques. In the second group of 10 manuscripts, all 3743 implants were performed percutaneously by physicians (not surgeons). In the 11 manuscripts in which the kind of physicians performing the procedure were not clearly indicated, a total of 5441 implants were reported; of these, 557 (10.2%) were performed with surgical cut-down techniques, and 4884 (89.8%) were performed by percutaneous techniques. Finally, 1 manuscript reported a total of 102 patients treated by radiologists in 49 cases (48.0%) by the percutaneous approach and in 53 cases (52.0%) by surgeons using the cut-down technique (Table 4).

**Table 4:** Types of physicians doing the procedure

Physician	Number articles	Articles with residents	Number implanted	%	Surgical technique	Percutaneous technique
Surgeon	15	4	8210	46.9%	2243	5967
Other	10	3	3743	21.4%	0	3743
Surgeon + other	1	-	102	0.6%	53	49
Not reported	11	-	5441	31.1%	557	4884
Total	37	7	17,496	100.0%	2853	14,643

Concerning the technique used in the 37 manuscripts, in 7 manuscripts (18.9%), surgical cut-down was reported, the percutaneous technique was reported in 23 articles (62.2%), and both techniques were described in 7 articles (18.9%). No US was used in the first 7 studies in which TIVAD was implanted with the pure cut-down technique. In the second group of studies, of the 12,399 patients undergoing percutaneous US, the vein was cannulated in 6636 patients (53.5%), while in 5763 patients (46.5%), the vein was punctured using anatomical blind landmarks.

Finally, in the third group of 7 manuscripts in which 2954 patients were treated with both techniques, the cut-down technique was used for 710 patients (23.8%), while the percutaneous technique was used for 2244 patients (76.2%). Among all of these patients, 1333 underwent US; for patients submitted to surgical techniques, the vein was located with US in 352/1333 patients (26.4%), while in the percutaneous technique group, the vein was localized by US in 981/1333 patients (73.6%) (Table 5). In the last two groups of manuscripts (23 manuscripts percutaneous only and 7 manuscripts with cut-down only), patients for whom the approach did not include US experienced 60 PNX (0.5%) in contrast to the patients in which the TIVAD was placed using US where 11 PNX were reported (0.09%) (Table 6).

**Table 5:** Number of patients in which ultrasound (US) was used to exactly localize the vein. Not US indicates patients in which the vein (both surgical or percutaneous) has been approached using anatomical landmarks

	Number articles	Number implanted	Surgical technique	Percutaneous technique	US	%	Not US	%
Surgical technique	7	2143	2143				2143	100.0%
Percutaneous technique	23	12,399		12,399	6636	53.5%	5763	46.5%
Both techniques	7	2954	710	2244	1333	45.1%	1621	54.9%
	37	17,496	2853	14,643	7969	45.8%	9527	54.2%

**Table 6:** Patients submitted to the percutaneous approach with (US) and without (NUS) US vein localization and related PNX

Percutaneous technique	12,399	%
US	6636	53.5%
PNX	11	0.2%
NUS	5763	46.5%
PNX	60	1.0%

Of the 2853 devices (16.3%) implanted by a surgical technique, 1822 devices (63.9%) were placed in the cephalic vein (CFV), 963 devices (33.8%) in the external jugular vein (EJV) and 68 devices (2.4%) in the brachiocephalic vein (BCV). Of the 14,643 implants (83.7%) placed with a percutaneous technique, 5784 devices (39.5%) were placed in the internal jugular vein (IJV), 5321 devices (36.3%) were placed in the subclavian vein (SCV), 2172 devices (14.8%) were placed in the axillary vein (AXV), 744 devices (5.1%) were placed in the innominate vein (INV) and 622 devices (4.2%) were placed in the brachiocephalic vein (BCV) (Table 7).

**Table 7:** Sites of the TIVAD implant

Total of implants 17,496			
Surgical procedure 2853	CFV	1822	63.9%
	EJV	963	33.8%
	BCV	68	2.4%
		2853	100.0%
Percutaneous procedure 14,643	IJV	5784	39.5%
	SCV	5321	36.3%
	AXV	2172	14.8%
	INV	744	5.1%
	BCV	622	4.2%
		14,643	100.0%

Legend: *CFV*, cephalic vein; *EJV*, external jugular vein; *BCV*, brachiocephalic vein; *IJV*, internal jugular vein; *SCV*, subclavian vein; *AXV*, axillary vein; *INV*, innominate vein

Immediate complications in patients undergoing surgical techniques were 32 (1.1%) HMMs. In patients treated with a percutaneous technique, the total complications were 333 (2.8%): 71 PNX (0.5%), 2 HMT (0.01%), 175 AAP (1.2%) and 85 HMM (0.6%).

No mortality was reported with either technique (Tables 8 and 9).

**Table 8:** Immediate complications that occurred with pure surgical techniques by author and type of complication

Year	Author	Surgical technique	PNX	HMT	APP	HMM
2010	Koketsu [11]	74	-	-	-	-
2011	Knebel [12]	53	-	-	-	1
2011	Di Carlo [15]	108	-	-	-	-
2011	Narducci [16]	771	-	-	-	26
2015	Lin [19]	27	-	-	-	1
2014	Cavallaro [20]	155	-	-	-	1
2014	Granziera [23]	102	-	-	-	-
2014	Cavallaro [24]	753	-	-	-	-
2015	Cavallaro [30]	83	-	-	-	-
2016	Otsubo [31]	149	-	-	-	-
2016	Zerati [32]	21	-	-	-	-
2017	Kagawa [36]	254	-	-	-	3
2019	Hashimoto [38]	203	-	-	-	-
2020	Mehta [45]	100	-	-	-	-
		2853				32

Legend: *PNX*, pneumothorax; *HMT*, haemothorax; *AAP*, accidental arterial puncture; *HMM*, haematomas

**Table 9:** Immediate complications that occurred with a pure percutaneous technique by author and type of complication

Year	Author	Percutaneous technique	PNX	HMT	APP	HMM
2010	Silas [10]	536	-	-	-	-
2011	Knebel [12]	49	2	-	-	-
2011	Lin [13]	113	1	-	0	-
2011	Barbetakis [14]	700	16	-	11	16
2012	Kim [17]	441	2	-	11	10
2013	Osawa [18]	207	3		1	
2015	Lin [18]	29	1	-	-	1
2014	Cavallaro [20]	143	2			2
2014	Cheng [21]	214	0	-	0	-
2014	Zhou [22]	492	-	-	6	12
2014	Granziera [23]	690	4	-	12	-
2014	Seo [25]	216	-	-	2	-
2015	Tagliari [26]	110	-	-	6	1
2015	Gurkan [27]	324	3	-	17	5
2015	An [28]	397	-	-	12	-
2015	Wu [29]	668	5	-	-	-
2016	Otsubo [31]	122	1	-	5	-
2016	Zerati [32]	1202	1	-	14	2
2016	Ma [33]	2996	9	2	-	4
2017	Feo [34]	527	3	-	-	-
2017	Seo [35]	932	-	-	-	-
2018	Hong [37]	176	-	-	-	-
2019	Hashimoto [38]	9	-	-	-	-
2019	Sun [39]	55	0	0	1	-
2019	Xu [40]	67	-	-	1	-
2019	Sun [41]	283	0	0	1	-
2019	Sun [42]	619	2	0	5	1
2019	Velioglu [43]	2084	16	-	63	27
2020	Souadka [44]	135	-	-	6	4
2021	Sun [46]	107	-	-	1	-
		14,643	71	2	175	85

Legend: *PNX*, pneumothorax; *HMT*, haemothorax; *AAP*, accidental arterial puncture; *HMM*, haematomas

There were 365 complications overall. In 10 articles (27.1%), there were 200 complications (54.8%), but they were not reported by the implantation site. In the remaining 27 articles (72.9%), it was possible to divide the complications by the implant site for a total of 165 complications (45.2%). With the surgical technique, HMM complications of TIVAD implantation were observed in the CFV 1 (0.1%), and there were cases of HMM after implantation in the EJV 4 (0.3%). With the percutaneous technique, the complications of TIVAD implantation in the IJV were 2 PNX (0.03%), 15 APA (0.3%) and 5 HMM (0.09%); with the SCV technique, the complications were 26 PNX (0.5%), 68 APA (1.3%) and 29 HMM (0.5%); with the AXV technique, the complications were 6 PNX (0.3%) and 5 APA (0.2%); with the INV technique, the complications were 1 PNX (0.1%), 5 APA (0.7%) and 1 HMM (0.1%); while with the CBV technique, there was 1 APA (0.2%) (Table 10).

**Table 10:** Complications in relation to the site of implant

Vein	Complications		
CFV 1721	PNX	-	
	HMT	-	
	AAP	-	
	HMM	1	0.1%
EJV 956	PNX	-	
	HMT	-	
	AAP	-	
	HMM	4	0.4%
BCV 68	PNX	-	
	HMT	-	
	AAP	-	
	HMM	-	
IJV 5784	PNX	2	0.03%
	HMT	-	
	AAP	15	0.3%
	HMM	5	0.09%
SCV 5321	PNX	26	0.5%
	HMT	-	
	AAP	68	1.3%
	HMM	29	0.5%
AXV 2172	PNX	6	0.3%
	HMT	-	
	AAP	3	0.1%
	HMM	-	
INV 744	PNX	1	0.1%
	HMT	-	
	AAP	5	0.7%
	HMM	1	0.1%
BCV 622	PNX	-	
	HMT	-	
	AAP	1	0.2%
	HMM	-	

Legend: *CFV*, cephalic vein; *EJV*, external jugular vein; *BCV*, brachiocephalic vein; *IJV*, internal jugular vein; *SCV*, subclavian vein; *AXV*, axillary vein; *INV*, innominate vein

## Discussion

When TIVAD was patented and used for the first time, the Seldinger technique was used all over the world for almost four decades [2]. However, the surgeon who patented the TIVAD does not use the Seldinger technique to implant it, and he prefers to surgically isolate a small and relatively peripheral vein, the cephalic vein. What was the rationale of the inventor of this technique to choose a surgical cut-down instead of a percutaneous approach? The most important implication that must be considered for the first implants is that at that time, only oncological patients needed a TIVAD. These patients were particularly frail because they had already undergone multiple vein punctures and they had experienced the local toxicity of anticancer drugs [47]. The Seldinger technique is complicated by PNX and hemothorax, which can be lethal. This was very likely the rationale for the choice of surgical approach, avoiding as much suffering and possible complications related to the Seldinger technique, among patients who already have a compromised quality of life due to their cancer. This rationale, which most likely was taken into consideration by J H Niederhuber, still remains, despite the widespread use of the Seldinger technique and should be one of the main concerns about the technical choice for TIVAD implants [48].

Over these 40 years, TIVAD has had great success around the world, and for several reasons (increases in indications for chemotherapeutic drug venous infusions, the inability of surgeons to satisfy massive requests for their placement, leading to an increased need for radiologists and anaesthesiology, etc.), and progressively, the cut-down technique has become less commonly used than the Seldinger technique. Unfortunately, the percutaneous approach has a risk of immediate complications, and if not coupled with US guidance, they can be serious or even lethal.

The Seldinger technique is an extremely useful venous line placement approach that is used in many fields of medicine. When it is used in cases of emergency or trauma, complications, especially PNX, can be justified because the procedure is being applied as an emergent lifesaving procedure. Different considerations arise when the patients needing the Seldinger approach are not in immediate danger and, as oncological patients, instead need a TIVAD placed avoid some complications related to chemotherapeutic drugs. In this last case, we have to guarantee better conditions, preserving the quality of life of these frail patients [49].

Despite these considerations in the last 10 years, the rate of immediate PNX related to the percutaneous approach is alarmingly high [8]. PNX, haemothorax and accidental puncture of arteries do not occur during the surgical techniques. Anatomical, landmark-based, “blind” percutaneous access for TIVAD implantation is associated with the majority of complications related to this percutaneous US technique. In contrast, when real-time ultrasound vein identification is used for vein cannulation, the results in terms of complications are improved. In fact, a 0.2% rate of PNX occurred when a systematic use of US guidance was chosen, compared with 1.0% of blind landmark percutaneous procedures (Table 6).

To preserve the safety of the surgical approach to the cephalic vein, a mixed technique has been described in the literature. These studies, excluded from the present study to avoid confusing data, are based on the possibility of using the Seldinger technique in part or in toto when the open cut-down fails. These techniques are associated with a nil PNX rate [50] and can be considered a valid rescue technique in cases of unusable cephalic veins. Another manuscript has reported cases of PNX after conversion from the cut-down to a percutaneous approach [51]. These studies, undoubtedly valid from the scientific point of view, are unacceptable for both patients who experience the complication and for surgeons, who may have been able to finish the procedure with a risk-free surgical approach [52, 53].

Although the classical cut-down technique is safe, failure of cannulation of the cephalic vein can occur [51]. Seldinger’s “blind” choice after an initial failure of the open technique must be absolutely avoided, and if clinicians want to use a percutaneous technique, US real-time guidance may be beneficial in such a situation. However, this revolutionary US technique does not fully prevent immediate complications.

Recently, another mixed technique has been reported [54]. In this manuscript, the authors report the complete surgical preparation of the internal jugular vein, and then after clamping, the catheter is inserted using the Seldinger technique through the venous wall. In this innovative procedure, however, there are 2 important complications to keep in mind: puncture of the carotid artery, which requires suturing, and the possibility of PNX. The focus of this manuscript is on immediate complications, but after preparation of the internal jugular vein, surgical insertion of the catheter may avoid these complications [54].

The best method to convert to in cases of failure of cephalic vein cut-down can be considered the technique of Knebel et al. [50]. In this case, the Seldinger technique is used in a hybrid form. In fact, the technique used involves introducing a guidewire into a stenotic vein and then tentatively dilating the same vein. If the procedure achieves its purpose, this represents the best method to complete the TIVAD implant without risk to the patients. In cases of failure of this technique, the direct Seldinger technique is not recommended and, instead, if the competencies of the physicians allow it, it is recommended to adopt real-time ultrasound vein cannulation. However, safety is only ensured by the surgical cut-down of another vein [52, 53].

TIVAD implantation is considered a simple procedure (both surgically and percutaneously), achievable by both senior residents and by surgeons. In the present study, manuscripts in which residents have been involved in both techniques have been reported. The rate of immediate complications with the percutaneous approach is near 0.05% [10, 13, 29]. In contrast, for the cut-down technique, the complication rate is nil [33, 34, 38]. Therefore, residents will have better success when they practice the cut-down technique.

The technique that is currently being used the most worldwide is the percutaneous approach. The major cause of this preference is related to the increased requests for a TIVAD that cannot be met by surgeons. In fact, the surgical approach usually requires an operating room, nurses and surgeons dedicated to the procedure. In contrast, when the procedure is performed by a radiologist or anaesthesiologist, it may be performed in a radiological interventional room with only two persons (a radiologist and a nurse).

This decreases the cost and time needed for the procedure, as well as the patient’s stress, who may think a procedure performed in an interventional radiological room is less risky and invasive than a procedure performed in an operation room. The same logistical situation can be related to the need for anaesthesiologists. These different situations have another consequence: the cost. The cost of a procedure and the related income are considered worldwide as one of the major factors to affect its acceptance by the administration, not only in nations where private/insurance system exists but also in countries where the taxes of citizens maintain a public health system, such as in Europe. The costs of the percutaneous approach are much lower than the surgical cut-down approach, and in addition, operation rooms can be reserved for more profitable surgical procedures [46]. The effort to control costs is increasing in all health systems around the world, frequently not in accordance with the aspects of the disease and the related quality of life of the patients. This aspect should be carefully re-evaluated by putting oncological patients first as targets of the situation, considering their disease and related quality of life as the primary objective of the health system before any economic considerations [55].

In the present review, the different veins seemed to require different techniques, with the cephalic vein best handled with a surgical approach and the internal jugular vein best handled with a percutaneous approach. The absence of immediate life-threatening complications when using the cephalic vein is quite acceptable. The internal jugular vein has a lower rate of immediate complications than subclavian vein access. Landmarks for the IJV are more evident and intuitive in relation to the subclavian vein, which can probably explain the difference between the two techniques when approached using landmarks. This represents almost all cases.

To avoid the majority of complications related to the puncture technique in the last 10 years, ultrasound techniques have been increasingly used. The aim of these diagnostic tools should be to eliminate the immediate complications related to the Seldinger technique, improve the quality of life of the patients and finally also influence the cost of the procedure. However, even with identification of the morphological position by US, the rate of PNX has decreased but is still higher than that for cephalic vein cut-down. Many reasons can be considered: (a) resident involvement in the procedure, who is not sufficiently trained and skilled to safely finish the procedure; (b) not all procedures are carried out by residents, and for skilled radiologists or anaesthesiologists, poor conditions of the patients (for example, obese patients) can be factors increasing the complications. A steep learning curve could be an additional factor affecting the complication rate.

US has also been used for investigating the status of the cephalic vein (their dimensions and deepness) [31, 38]. This technique can be useful for the diagnosis of an anatomical absence of this vein. In this case, the EJV can be approached surgically [53]. In contrast, in the case of an US diagnosis of a narrow cephalic vein, the approach to this vein is not prohibitive because it can be dilated and cannulated using a mixed technique. No scientific recommendation exists in the literature for cases where it is impossible to use the cephalic vein, but the surgical use of another nearby vein can preserve the patients from complications [8, 52, 53].

Arterial puncture is particularly related to percutaneous techniques, and this complication can be fatal if associated with haemopneumothorax [56]. This complication is still present, both before and after US technique applications, and can cause a haematoma when the Seldinger technique is applied. This complication can also occur when using a cut-down technique, but in this case, the cause is related to difficult haemostasis for coagulation disorders or a difficult or incorrect dissection, especially in obese patients. In the present review, haematomas, as a complication of surgical cut-down, are presented in a manuscript in which almost all cases were reported. In this manuscript, both the cephalic vein and the external jugular vein were used, but there was no specification about the most frequently involved vein or the cause of the high rate of haematomas. The only factor that can be considered is that the majority of the procedures were performed by residents. However, this cannot be proven to be the cause for sure.

In this review, in contrast to the previous one [53], no mortality was reported. This is due to an ever-greater experience in most institutions with this procedure, preventing this final complication.

## Conclusions

The percutaneous approach is currently the most commonly used technique to implant a TIVAD. Over the years, many methods have been used to decrease immediate complications that can be fatal, such as PNX or haemopneumothorax. Employing US to localize the vein well and to guide vein puncture is the most popular and strongly recommended method. The incidence of PNX has decreased in the last 10 years when US is used for the percutaneous approach, but despite the many efforts, immediate complications are still occurring. Surgical cut-down remains the only technique that can avoid all the immediate complications 40 years after the first implant, especially those that can be fatal for the patients.

## Data Availability

Not applicable.
